# “Breastfeeding at Night Is Awesome” Mothers’ Intentions of Continuation of Breastfeeding Extreme and Very Preterm Babies upon Discharge from a Kangaroo Mother Care Unit of a Tertiary Hospital in South Africa

**DOI:** 10.3390/healthcare11071048

**Published:** 2023-04-06

**Authors:** Sphiwe Madiba, Perpetua Modjadji, Busisiwe Ntuli

**Affiliations:** 1 Faculty of Health Sciences, University of Limpopo, Polokwane 0700, South Africa; 2 Non-Communicable Disease Research Unit, South African Medical Research Council, Cape Town 7505, South Africa; 3 Department of Public Health, School of Health Care Sciences, Sefako Makgatho Health Sciences University, Pretoria 0208, South Africa

**Keywords:** kangaroo mother care, breastfeeding, intensive neonatal care, preterm infants, very low birth weight, South Africa

## Abstract

Kangaroo mother care (KMC) is effective in increasing mothers’ initiation and maintenance of breastfeeding (BF) for extreme and very preterm (VLBW) infants. Although South Africa has implemented KMC for more than two decades, little is known about mothers’ perspectives on KMC. The purpose of this study was to describe the BF intentions and practices of mothers of VLBW infants at home following discharge and assess the role long stay in KMC has on their decision to BF beyond discharge. This qualitative study was conducted at the KMC unit of a tertiary hospital in Pretoria, South Africa. Focus group interviews were conducted with 38 mothers of VLBW infants who had transitioned from neonatal intensive care (NICU) to KMC. We analysed transcripts following the five steps for qualitative thematic data analysis. Mothers were knowledgeable of the importance and value of BF preterm infants and conceded that breast milk has advantages over formula. Mothers had positive feelings toward BF their preterm infants. The stay in KMC increased the direct BF of their preterm infants, mothers’ BF efficacy, and had a positive influence on mothers’ intentions to continue BF following discharge and to exclusively breastfeed for six months. Their BF intentions, efficacy, and practices were influenced by the skilful BF counselling, training, and support they received from the nursing staff. High intention to BF among these mothers is suggestive of their knowledge and confidence in BF for their VLBW infants. It is important that nursing staff in NICU and KMC appreciate the significant role they play in mothers’ readiness and confidence to breastfeed beyond discharge.

## 1. Introduction

South Africa has implemented kangaroo mother care (KMC) for more than two decades [1] as a care model to strengthen the family bond, encourage breastfeeding, and promote safe hospital discharge for preterm babies and their families [2]. KMC is an intervention aimed at improving outcomes among preterm and low-birth-weight new-borns. The core components of KMC include early continuous and prolonged skin-to-skin contact between the mother and the baby, and exclusive breastfeeding (EBF) or feeding with breastmilk [3]. In addition to the three components of KMC, South Africa and other countries added a fourth component of kangaroo care that includes emotional, physical, and educational support given to the mother to meet her infant’s needs whilst practising KMC in the hospital and beyond [4]. 

The KMC method is a public health policy with several health benefits for the mother–child dyad. KMC is important for the promotion of EBF during the hospital stay and has been linked to an increase in breastfeeding (BF) rates and the duration of BF of preterm very low birth weight (VLBW) infants [2,5]. The World Health Organisation (WHO) classifies preterm infants based on gestational age as extremely preterm (<28 weeks), very preterm (born between 28 and 32 weeks), and moderate to late preterm (born between 32 and 37 weeks) [6]. The health benefits of BF for VLBW infants can be characterised as having short- and long-term outcomes [7]. BF short-term benefits include lower rates of necrotizing enterocolitis, rehospitalisation, healthcare-associated infection and sepsis, and overall mortality [8,9,10], while long-term benefits include increased survival, early weight gain and improved growth, neurodevelopment, and infant–parent bonding [7,11,12]. The results from a systematic review and meta-analysis have shown that KMC certainly has positive effects on the growth and BF rates in VLBW infants [4,8].

In previous studies, it was established that preterm infants often have a delayed initiation of BF compared to full-term infants [13,14]. In addition to the physiological reasons, such as difficulty with latching, sucking, and swallowing due to their immaturity, separation of the mother from her VLBW infant contributes greatly to the delayed initiation of BF [2,6,14,15]. There is significant evidence that VLBW babies are at the highest risk of lower rates and a shorter duration of EBF than term infants [16,17]. Furthermore, only a small portion of mothers of preterm infants use EBF [18]. Research further reports that mothers with limited BF during NICU admission experience many challenges to BF their preterm infant after discharge [19,20].

Whereas the initiation of BF in the NICU is challenging, discharge from the hospital is more critical regarding the continuation of BF for VLBW infants [18]. Even though mothers practise feeding their VLBW infants during the KMC stay, the transition to home can be challenging, and after returning home, breastfeeding is a major challenge [16,21]. Whereas mothers long for discharge, during the stay in the neonatal or KMC unit, they adapt to the hospital routines. However, discharge and the responsibility to provide care for their infants on their own cause them uncertainty [16,22]. Adequate discharge preparation can help mothers of preterm infants successfully transition from the KMC or neonatal unit to the home [23]. Therefore, it is crucial that, after discharge from hospital, mothers feel that they can provide safe care to the preterm infants at home [24]. 

A recent integrative review of the literature corroborated previous findings that the quality of discharge teaching is a strong predictor of discharge readiness [22]. Previous studies have established that successful transition from the KMC or neonatal unit to the home is fundamental for the long-term health and well-being of preterm infants [16,22,25]. The mother’s discharge planning increases the mother’s readiness for the infant’s transition from hospital to home [26,27]. In addition to a supportive environment in the KMC and at home, special efforts from the mother, her motivation, and belief in BF are essential for successful BF [6,19,28]. It is therefore evident that the mothers’ BF self-efficacy is key to the establishment and maintenance of BF in the short and long term [2,29]. BF self-efficacy encompasses the mother’s ability and confidence to successfully BF and has been identified as a protective factor against short BF duration [30].

Although data from previous studies emphasise that KMC is effective in increasing mothers’ initiation and maintenance of BF during the hospital stay [2,8,31,32,33], there is a dearth of this kind of research and data in South Africa. Furthermore, the focus of previous studies conducted elsewhere was on mothers’ experiences of BF VLBW infants during their stay in KMC or in a neonatal unit [34]. Moreover, the focus of studies after discharge is on the duration of BF VLBW infants after discharge from the hospital [15,21,35]. It is generally acknowledged that successful transition from the KMC to home, achieved through education and support, is fundamental for the long-term health and well-being of VLBW infants [16,22,25]. However, little is known about the mothers’ perspectives on the KMC discharge process and transitional care experiences [25], as well as mothers’ future BF plans for their VLBW infants after discharge from the hospital [13], particularly in low- and middle-income countries, including South Africa. 

The purpose of this study was to describe the breastfeeding intentions and practices of mothers of VLBW infants at home following discharge after a long stay in a KMC unit. Although studies emphasise the positive effect of KMC on BF such that the stay in KMC increases self-confidence of mothers in both feeding and caring of infants [31,32], studies examining how KMC affects the perceptions of mothers concerning BF are limited [33]. This is particularly true of South Africa, even though educational support is given to the mother on how to meet her infant’s needs with the focus on BF while practising KMC in the hospital and beyond [4]. 

Researchers have established that skilful BF counselling and enhancement of mothers’ motivation by nurses is essential while mothers are practising KMC [18,28]. Therefore, a secondary aim of the study was to assess the impact the long stay in the KMC unit has on mothers’ decisions to BF beyond discharge. The authors of [33] suggest that that mothers who have high-level perceptions of effective BF would be successful during the breastfeeding journey.

Examining the mothers’ BF plans for their preterm infants after the hospital stay will provide a new perspective which health providers could use to develop BF support during the stay in the KMC unit in preparation for discharge and the sustenance of BF duration at home [18]. Understanding the effect of KMC on mothers’ perceptions regarding breastfeeding will guide healthcare professionals through their practices and education [13].

## 2. Materials and Methods

### 2.1. Study Design

This qualitative study was conducted at the KMC unit of a tertiary hospital in Pretoria, South Africa between September, and November 2015. Participants consisted of mothers of VLBW infants who had been hospitalised in the NICU and had been transitioned to the KMC unit where they room-in with their mothers. When the preterm infant is admitted in NICU, the mothers are housed in three lodger wards in the hospital. This allows the mother to express breastmilk to feed the preterm infant through tube feeding while they are admitted in NICU. The infants are discharged from NICU when they are medically stable. During rooming-in in the KMC unit, the mothers continue tube feeding their infants every two hours under the guidance and assistance of the nurses. When the infant is ready, the mother transitions to direct BF, and nurses monitor the weight of the infants in preparation for discharge. Pre-term infants are discharged from KMC when they reach a weight of 1800 g and their suckling reflexes are well developed. Detailed data on KMC practices and procedures are described elsewhere [36]

### 2.2. Study Setting and Population

**Figure 1:**
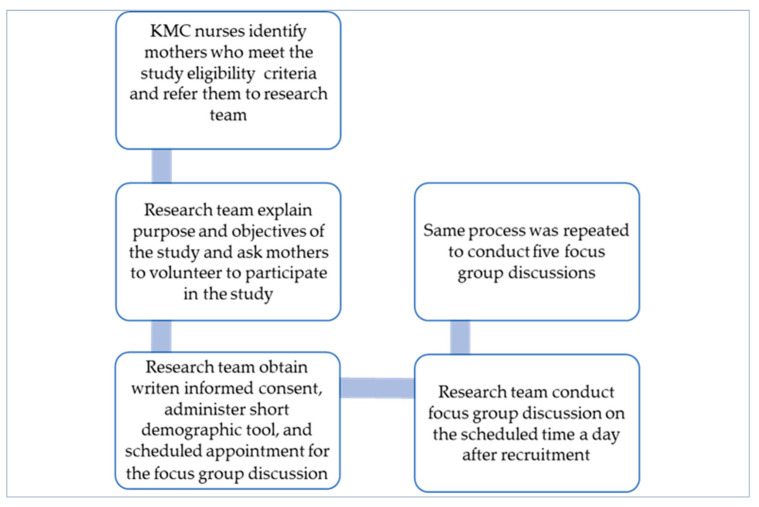
Schematic presentation of recruitment and data collection.

The KMC unit can accommodate up to 30 mothers at a time, and there is a continual flow of mothers and pre-term infants as they are admitted and discharged from the unit. We therefore had a pool of mothers to recruit during the study period. We selected mothers who had begun to BF their preterm infants through a purposive sampling technique. The KMC unit manager informed the mothers about the study, and nurses working in the KMC unit identified and approached potential participants who met the inclusion criteria. Those who were interested were referred to the research team, which explained the purpose of the study in detail (Figure 1).

### 2.3. Data Collection, Tools, and Procedure

Data were collected through focus group interviews, which took place in a private area in the KMC unit, in the afternoon as the unit was at its quietest then, owing to the completion of doctors’ and nurses’ rounds. The focus groups were moderated by a research team trained in qualitative research methods. A focus group guide based on the literature on BF preterm babies [13,37], comprised of open-ended questions, was used to explore the mothers’ perceptions about KMC and their intentions to BF beyond discharge. Probes were used as needed to explore the topic in greater depth, to elicit more information, and to clarify the responses provided by the participants. 

The focus groups were conducted in the local language, Setswana, after written consent had been obtained from the mothers. All the participants’ responses were audio recorded, and each session lasted approximately one hour. Data saturation was reached after five focus groups had been conducted with about 7–8 mothers in each group. Data saturation was achieved when subsequent discussions no longer generated new information to contribute to the understanding of the participants’ experiences of breastfeeding their preterm infants [38].

Participants’ demographic information, such as their age, marital status, employment, educational attainment, and support system, was captured through a demographic tool. In addition, obstetric and clinical data, such as the participants’ parity, number of pregnancies, gestational age, infants’ birth weight, current weight, length of stay in the ICU, and length of stay in the KMC unit, were collected.

### 2.4. Ethical Considerations

The MEDUNSA Campus Research Ethics Committee (MREC) of the University of Limpopo (MREC/H/81/2014: PG) provided ethical clearance of the study. The relevant hospital authorities granted permission to conduct the study. All mothers provided individual written informed consent prior to the interviews, were informed that their participation was voluntary, and were told about the confidentiality of the study. We used synonyms during the interviews to protect the identities of the mothers, as well as and when we present direct quotes to provide context of the findings.

### 2.5. Data Analysis

Qualitative thematic analysis was the approach used to analyse the data, using NVivo 10. The research team transcribed audiotaped data verbatim and translated transcripts into English. Braun and Clarke’s [39] six steps for qualitative data analysis guided thematic analysis approach, which allowed for both deductive and inductive analysis. First, we read the transcripts several times so that we could familiarise ourselves with the data and identify initial emergent codes. We then searched for themes; reviewed, defined, and named emerging themes; grouped the codes generated from the transcribed interviews into themes and sub-themes; and developed a coding frame. We continued to reconcile the emerging codes and themes until deep and rich themes and sub-themes that described the mothers’ feelings about BF preterm infants and intentions to breastfeed beyond discharge had been achieved. Direct quotations from the participants were used to support the themes.

### 2.6. Rigour

Rigour in this study was obtained through several techniques, and trustworthiness was ensured through the principles of credibility, confirmability, dependability, and transferability [40]. The focus group discussions were conducted in the local language and transcribed verbatim to enhance the credibility and dependability of the data. Confirmability was achieved using field notes and audit trails. All authors analysed the data and participated in the discussion about the final themes and sub-themes to ensure that the interpretations were free from investigator bias. In addition, we used NVivo qualitative software to analyse the data [41].

## 3. Results

### 3.1. Sociodemographic Profile of Participants

The sample included 38 mothers of preterm infants in the KMC unit. At the time of interview, the mothers’ length of stay in the hospital (NICU and KMC) ranged from 7 days to 12 weeks. All the mothers were successfully BF their infants except for two mothers whose infants were transferred to KMC a day before the interviews and had not been able to latch nor suckle properly. Maternal age ranged from 16 to 40 years, with a median age of 27 years. Only four mothers had obtained tertiary education, 16 had completed high school, 15 had obtained a secondary education, and three had primary education. As to parity, 10 of the mothers were primiparas and 28 were multiparas. Of the multiparas’ mothers, 18 had been pregnant twice, three have been pregnant thrice, and seven had been pregnant 4 times and more. The number of pregnancies ranged from 1 to 6. None of the mother had a previous history of a preterm birth. Of the 38 mothers, 14 had one child, 15 had two children, and nine mothers had 3 children and more. The number of children ranged from 1 to 5, with a mean of 3 children. 

The gestational age of the Infants ranged between 24 and 36 weeks, and the mean gestational age was 29 weeks. The infants’ birth weight was between 660 and 1800 g, and the mean birth weight was 1303 g. The sample included extremely, very, and moderate to late preterm infants. The infants had been in the NICU from 3 days to 6 weeks, and the mean length of stay in the NICU was 14 days. At the time of the interviews, the infants had been in the KMC unit from 1 day to 8 weeks, and the mean stay in KMC unit was 12 days. The infants’ current weight was between 840 and 1800 g, with a mean weight of 1556 g.

### 3.2. Themes

**Figure 2:**
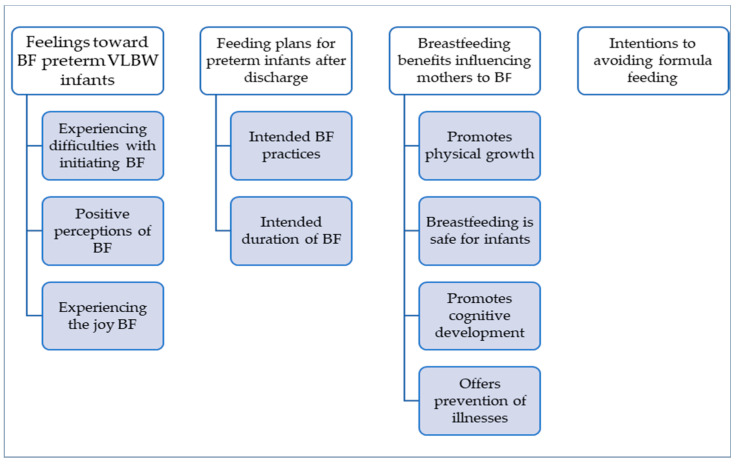
Summary of the themes and supporting sub-themes.

The four main themes and supporting sub-themes that emerged from the data analysis about feelings about BF VLBW infants and the intention of mothers to breastfeed infants beyond discharge are displayed in Figure 2.

#### 3.2.1. Feelings toward Breastfeeding VLBW Infants

At the time of the interviews, all mothers except for two were successfully BF their infants, and all the infants were no longer in the NICU. The infants of the mothers who were not BF had not been able to latch yet because they were discharged from NICU a day before the interviews. When the infants were admitted to the NICU, they were tube-fed breastmilk. Once the infants’ sucking reflex had developed enough and they were discharged from NICU, mothers were told to commence direct BF. Three supporting themes—experiencing difficulties with initiating BF, positive perceptions of BF, and experiencing the joy of BF—describe mothers’ feelings toward BF preterm infants during their stay in KMC. 

Experiencing difficulties with initiating BF:

When mothers transitioned form tube feeding to direct BF their preterm infants, they reported that BF was difficult. They experienced BF as scary and uncomfortable, especially mothers of extremely and very preterm infants. 

*It was stressful to breastfeed him because his mouth is small, and the nipple is big. You have to keep forcing things so that he can at least suck a bit. He is not like a full term baby (Nthabiseng, 23 years old).*

*I saw it as though this little mouth was too small. When she sucks, I saw it as though she is feeling pain, she was too small (Portia, 28 years old).*

*I was scared… I was scared they (twins) were too small (Sophie, 22 years old).*

The experience was filled with uncertainty about whether the infants were getting enough milk or not because of their small stature.

*I used to ask the nurse that: “Sister, when she is sucking on this breast, is she getting milk? But, this stomach, is it getting full? Is she getting full?” (Mpho, 20 years old).*

When transitioning to BF, mothers encountered several problems. They expressed frustration with infants who would not latch or suckle successfully, infants sleeping for long hours, and those who were difficult to awaken for feedings.

*I still have a problem with her being able to suck on my breast. The milk is there, but she is struggling to latch on to the breast. She sleeps a lot and, my nipple is big… It is a bit challenging (Thabang, 28 years old).*

*When I was going to breastfeed her sometimes, she would sleep. Breastfeeding is problematic, the babies sleep (Mpho, 35 years old).*

Mothers who initiated breastfeeding shortly before the interviews described painful experiences. They reported that breastfeeding hurt and caused discomfort for them. 

*Breastfeeding is painful, I feel a lot of pain. It is very painful to breastfeed… I was happy to be expressing, but now that she is breastfeeding directly, it is very painful (Asiphe, 22 years old).*

*When the baby does not start breastfeeding and is tube fed for a long time, they struggle to learn how to suck and when she has to suck on the breast for the first time, she becomes slow. So, because of this, we stay in the hospital for even a longer time, while I teach her how to suck time (Dimakatso, 22 years old).*

Since mothers were expected to express milk when their infants were in the NICU to maintain milk secretion, some reported that they found expressing breast milk more comfortable than direct BF. 

*I was happy to express… When I was expressing breast milk for her, I was just relaxed, I didn’t have any stress, and I didn’t have any problems. I was just concerned about my baby being calm, her eating and getting full. Now that she is breastfeeding directly, it is very painful (Asiphe, 22 years old).*

Positive perceptions of BF:

Whereas some mothers could transition to BF in a short time without problems, others took much more time and experienced direct BF as a challenge. However, with time, for most of the mothers, the negative feelings toward BF extremely and very premature infants changed to more positive ones as they experienced their first BF.

*I was happy when I heard that she would start breastfeeding. I was happy because I knew that with the breast, she would have to suck by herself with the mouth and that I would not be giving her with the tube because she sleeps during tube feeding, at least with breastfeeding she knows that she has to wake up and suck (Ivon, 28 years old).*

*Breastfeeding my baby was nice because I saw that when she stopped tube feeding and started breastfeeding, she was indeed growing and that she could suck on the breast by herself. This proved to me that she would be able to do anything by herself. She is sucking well now (Sindi, 36 year).*

*Breastfeeding didn’t give me a problem. When the nurses said that I should put him on the breast I was thinking that he is small to breastfeed, but when I put him on the breast, he opened his mouth, he started sucking and never gave me a problem. Actually, after putting him on the breast, that’s when he started gaining weight (Thandiwe, 28 years old).*

Although mothers experienced pain during BF, they felt fulfilled knowing that their milk was helping their infants grow.

*It was painful at the start, but I told myself that I am doing it for my baby. And it wasn’t a lot, it was a little, but I told myself that as long as my baby eats and gets full. So, I became okay and now the milk is coming out and it is no longer painful (Sophie, 22 years old).*

Mothers with late preterm babies perceived BF as a natural healthy process that could be compared to BF a term baby.

*With breastfeeding her I felt that it was the same as breastfeeding a 9 month old baby because there is no difference when you breastfeed her (Sindi, 36 years old).*

*Breastfeeding my baby for the first time taught me that I should accept that my baby is the same as those who came at 9 months, there is no difference (Sesie, 32 years old).*

*She is getting full, you are going to see her taking the breast out, not wanting it anymore and you… She will be right (Mpho, 20 years old).*

Experiencing the joy of BF:

After their preterm babies were discharged from the NICU, the mothers reported that they started to experience the joy of BF. They treasured the time they spent BF their infants because it promoted mother–baby interaction, and they expressed the joy of bonding with their babies. 

*Yoh, breastfeeding for the first time felt nice because it meant that I would bond with my baby while I am breastfeeding her and looking at her (Lethabo, 25 years old).*

*When you keep giving her that breast, you bond. You have that connection of mother to child (Ayanda, 27 years old).*

*I only know one most important benefit of breastfeeding, bonding with your baby so she can know you (Busi, 24 years old).*

*When I started to breastfeed him, I felt that the contact between me and him would now be easy. I look at him in the eyes while I am breastfeeding him and accept him like any other baby that came on time (Sesie, 32 years old).*

#### 3.2.2. Feeding Plans for Preterm Infants after Discharge

Most of the mothers expressed that their plans were to continue BF their preterm infants after they were discharged from the KMC unit. Their narratives revealed that the decision to BF after discharge was informed by what they were taught by the nurses in the KMC unit.

*My plans are that I will continue breastfeeding, even though I am going back to work, I will keep on expressing breast milk and leave it at home (Thabang, 28 years old).*

*When I get home, I am going to continue giving him the breast, until he is a bit older (Thando, 37 years old).*

*Indeed, I want to continue with breastfeeding, there’s no way I can stop (Monica, 23 years old).*

*Breastfeeding is it. Indeed, I am going to continue with it… (Angie, 21-year-old mother).*

*I am going to continue breastfeeding my baby together with expressing breast milk for her (Suzanne, 36 years old).*

Intended BF practices:

When the infant was admitted to the NICU and later to the KMC unit, mothers were trained in feeding their preterm infants. When the infants were initiated on tube feeding, the mothers were trained on how to feed their infants through the tube. They were also shown and supported to express breast milk to be used during tube feeding. When the infants had developed well and transitioned to BF, the mothers were trained on how to breastfeed. The narratives from the mothers indicated that they planned to continue with the infant feeding routine and patterns that were practised in the KMC unit after discharge from the hospital. Mothers breastfed their infants according to the two-hour schedule while the infants were admitted to the NICU and throughout their stay in the KMC.

*My feeding plans are to continue feeding my baby exactly as we were taught here at the hospital. They taught us about the times when to breastfeed our babies. When we are in here, the sisters wake us up to feed the babies, so because of that our bodies are already used to it. I will also breastfeed my baby on demand, because in the KMC Unit, we breastfeed them on demand (Tebogo, 22 years old).*

*I don’t think that I will change anything. I am going to continue breastfeeding him like this because this is my first baby. I can’t start by changing things, he might surprise me going forward, he might change and not become the way he was when he was breastfeeding (Mmatsheko, 16 years old).*

*My feeding plans won’t change. Just as how they taught us here in KMC, they won’t change. I am going to continue breastfeeding my baby. I also want to thank KMC because it taught us the importance of breastfeeding. Now I can see that baby gets full, from just breastfeeding her alone, I don’t have to supplement her with formula milk as I had thought previously. So KMC has taught us well (Portia, 28 years old).*

On the contrary, a few mothers stated that they would not adhere to the feeding patterns they had established in the KMC unit. They expressed that the times and routine were strenuous, and they would not cope at home where they would have to go back to work or school.

*I think that the times they give us in here are strenuous. Every 3 h, what about the mother? You don’t rest, you give the baby the breast while you are sleeping sometimes, you are not safe with the baby, and the baby might choke. They don’t give us a chance to breastfeed our babies willingly with love. You find that you are breastfeeding the baby while you are thinking: “But, I am tired” (Dimakatso, 22 years old).*

*I don’t promise to stick to the times that we were using in the ward to feed our babies. I have a strenuous job and I am busy studying, so waking up during the same times is going to be a problem (Suzanne, 36 years old).*

*I don’t agree with the feeding times we are using in here because when it’s time to feed her, you find that she is still full of the previous feeding time and if you force her to feed, it causes her to vomit, and her weight will drop. So, when I am at home she will cry when she wants the breast and I will feed her on demand (Ivon, 28 years old).*

Intended duration of BF:

A mother’s positive attitude toward BF plays a critical role in both the establishment and duration of BF preterm infants during the stay in the KMC and at home after discharge. The mothers’ narratives revealed their intentions to exclusively breastfeed their infants for six months as it was beneficial for their preterm infants. The plans to practice EBF were influenced by the teachings of the nurses in the KMC unit.

*I am also going to try to continue with breastfeeding for a period that the nurses say is accurate for a premature baby to have breast milk. I will breastfeed until my baby is six months, even if I am going back to work (Daisy, 29 years old).*

*I am going to continue with breastfeeding until the baby is six months, there’s no way I can change… I plan on breastfeeding (Angie, 21 years old).*

*I will continue with breastfeeding for 6 months, then only after that I will introduce other foods (Busi, 24 years old).*

Amongst the mothers, there were those who reported that they would like to achieve the required duration of two years of BF their preterm infants.

*Not only am I going to continue with exclusive breastfeeding until six months, but I will breastfeed until the baby is 2 years (Pinky, 31 years old).*

*I will continue to breastfeed him until he is 2 years old, the nurses say so (Maria, 19 years old).*

*I am going to continue breastfeeding her until she is 2 years old. In that way I will be satisfied that she is healthy (Sindi, 36 years old).*

#### 3.2.3. Breastfeeding Benefits Influencing Mothers to BF 

The mothers’ narratives reaffirmed the importance of BF to the baby and mother. Their narratives suggested that they were well-informed of the importance and the benefits of BF.

Promotes physical growth:

*I won’t change breastfeeding because they are [twin babies] my first children…, I won’t change because I see breastfeeding as an important thing for these babies so they can grow and become healthy (Mpho, 20 years old).*

*My feeding plans are to continue feeding my baby exactly as we were taught here at the hospital. So, when you breastfeed a baby, she grows up quickly, she becomes clever even at school (Tebogo, 22 years old).*

*I will continue to breastfeed him until he is 2 years old, the nurses say so. When you breastfeed, you know that he has eaten enough and you can see how he is growing (Maria, 19-year-old mother).*

*Breastfeeding is safe for infants*

*It’s awesome to feed at night because if you are sleepy, you can just take the breast out and give it to the baby. Breastfeeding is number one (Nthabiseng, 23 years old).*

*I am going to continue breastfeeding her the way that we were breastfeeding them because the sisters told us that the breast does not have an overdose, you just give it to her when she wants it. She will show you when she has had enough, she will stop sucking on the breast (Lebo, 39 years old).*

Promotes cognitive development: 

*How the nurses teach us here, I am going to continue breastfeeding in the same manner at home. When I breastfeed, she will develop quickly mentally (Lethabo, 25 years old).*

*For me the best thing to do is to breastfeed, when my child grows up, her mind is going to work well because she sucks the breast that comes from her mother. It helps the baby’s memory work fast (Mpho, 35 years old).*

Offers prevention of illnesses:

*I want to breastfeed her only rather than formula feeding her because when I breastfeed her, she can grow and prevent illnesses, she won’t get sick easily (Lerato, 19 years old).*

*Formula milk is not good for babies, it causes constipation. So, I prefer breast milk because it is a medicine. Also, breast milk helps the baby gain weight quickly, even when she is sick, she can fight against the illness compared to when she has formula milk (Ivon, 28 years old).*

Intentions to avoid formula feeding:

The mothers stated that they would avoid formula feeding because BF was a safer and more beneficial feeding option than formula. Their narratives suggested that they would never have to give formula because of the teachings they received from the nurses on the disadvantages of formula feeding. 

*Before I came to KMC, I preferred giving my babies formula milk with the bottle. I thought that it as being the best. When I arrived at KMC they taught us that giving the baby formula delays the baby growing and causes them to have constipation and other illnesses. They say that you need to have a safe place to put your bottle, so insects don’t crawl on it, like a sterilizer because in our cupboards there are cockroaches, flies in the house, dust and things like that. We don’t have anywhere safe to put them in our houses. We don’t always have the money to afford sterilizers, and having things like that becomes an effort, so the best thing to do is to breastfeed (Mpho, 35 years old)*

*I am going to continue with what the nurses have taught us, to wash our hands…, that whatever you do, you have to wash your hands before you breastfeed the baby, that is how the sisters have taught us (Gontse, 26 years old).*

They especially had concerns about hygiene issues related to preparing formula milk, as well as the disadvantages of formula feeding, such as infant weight loss or slow weight gain and diarrheal diseases.

*I see the breast as a very important thing, you can breastfeed instead of giving bottles or cups because a lot of germs enter through those things (Sindi, 36 years old).*

*The bottle is not good because I touch it with my hands and breast, I hold it without touching the milk. It won’t matter how I hold the breast; it will go straight into the baby’s mouth. When you keep touching the milk in the bottle, you won’t know if you washed your hands properly. So, the breast doesn’t need to be washed, it is okay the way it is (Sophie, 22 years old).*

*I don’t support formula milk because I work. I could handle my baby’s bottle in a clean manner. and you find that the person who is staying with the baby might only rinse out the bottle before making milk with it for the baby instead of washing it thoroughly. Formula milk is not even on my mind (Sesie, 32 years old).*

## 4. Discussion

This study involved mothers of VLBW infants in a KMC unit of a tertiary hospital in South Africa. Mothers described their feelings about BF their VLBW infants as positive and negative experiences. Though BF has been identified as especially difficult and complex in VLBW infants [18,34], the mothers’ narratives indicated that BF their preterm infants worked out well for many of them. We found that once those who had previously expressed negative perceptions of BF had gained more experience and were more comfortable with BF their preterm infants, their perceptions changed to being more positive. They experienced BF as pleasurable and appreciated the advantages that it entailed for their infants. This phenomenon was reported in previous studies where it was also found that, with time, mothers overcome the difficulties and stressful negative feelings, and BF becomes well-functioning and pleasurable [24,34]. 

Mothers experience BF negatively since the infants’ prematurity complicates feeding. Similar to prior studies, not being able to breastfeed their infants immediately after delivery evoked a wide range of feelings for the mothers [24]. The initiation of BF was daunting for the mothers, as they experienced frustration when their infants had weak suck, did not suck, or did not want to breastfeed. Previous studies reported similar problems of BF preterm infants [34]. Moreover, mothers were fearful of BF their preterm infants because of their stature; they felt they were so small, delicate, and helpless [24]. 

We found that the mothers in this study were knowledgeable of the value and the benefits of BF. They reaffirmed the importance of BF preterm infants [24]. Consistent with what has been reported in other studies, the mothers described BF as important for forming attachment and bonding with their infants [13,34]. They stressed the role that BF played in allowing their infants to recognise them, thereby helping in forming an attachment [13]. They viewed BF to optimise their preterm infants’ health because of its high nutritional benefits; ability to enhance their immunity, prevent illnesses, and promote optimal growth; and high economic value [13,24]. 

It was evident in this study that the mothers’ stay in the KMC unit increased the direct BF rate of VLBW infants. All mothers whose infants were discharged from the NICU were BF except for two mothers whose infants had spent only one day in KMC. Prior studies have described the role of KMC in promoting the mother–infant contact and bonding, success at BF, as well as the duration of BF for VLBW infants [4,20,24,42]. Research has established that the mother–infant bonding for preterm infants is initiated with the first direct BF in either the rooming in facility or KMC unit. Therefore, it is essential that direct BF is commenced as soon as possible for all preterm infants who are clinically stable [24]. 

The mothers in this study were confident that they would provide the care their preterm infants needed after discharge. Research has shown that direct BF in the NICU and KMC prepares mothers for BF after discharge [43]. Similarly, improved BF success is evident among mothers who put their preterm infants to their breast during the hospital stay [44]. As already indicated, the mothers in this study stayed for a long duration in the KMC and received counselling and training on BF from nursing staff. Previous research has reported on the positive contribution of nursing staff training for successful BF [45]. Mothers in a study conducted by Gianni and colleagues indicated that BF support by nursing staff is crucial for successful BF [46]. In another study, mothers of preterm infants associated adequate nursing support with better BF outcomes [14]. Other factors that influence BF preterm infants include the positive attitudes and clear breastfeeding goals of the mother while practising KMC [43]. 

The mothers in this study intended to feed their babies exclusively with breast milk up to the age of six months, following the two-hour feeding schedule they practised in KMC. Mothers reported that their decision was influenced by what they had been taught by the nurses. They stressed that, since everything that they were taught in KMC worked well for their infants, they intended to continue and would avoid any changes that might affect the growth of their infants. Previous studied have reported mothers’ observance of instructions they received from nursing staff [24]. We found that the transition from tube feeding to direct BF varied for every preterm infant, and the weight and clinical condition of the infants determined the transition to BF. Nevertheless, at every stage of the feeding journey, the mothers received the requisite support, training, and counselling from the nursing staff. Of particular importance was how to perform KMC, the times when to BF, and how to position their infants for BF. Like previous findings, high intention to breastfeed among these mothers is suggestive of their knowledge and confidence in their ability to BF their VLBW infants [21]. 

The mothers conceded that breast milk has advantages over formula. They indicated that they especially preferred BF over formula feeding after the nurses in the KMC unit taught them about the benefits of BF their preterm infants. Of importance is that mothers had witnessed first-hand their preterm infants gaining weight and being satisfied with breastmilk alone. Their narratives indicated that they would never have to give formula because they have an adequate milk supply and were uneasy about cleanliness related to preparing formula milk. Moreover, they understood the disadvantages of formula feeding, such as infant weight loss or slow weight gain and diarrheal diseases. Hence, they had intentions to exclusively breastfeed their preterm infants. Mothers in other studies have also believed that BF was the best for the infant and was beneficial for the infant’s health [45].

The transition to home, however, can be challenging for mothers of preterm infants, even if BF is well established [21]. Research has reported that mothers experience many challenges to BF their preterm infants after discharge. There is evidence of a steady decline in mothers who continue to breastfeed their preterm infants following discharge from the hospital [13,19,20]. Therefore, it is important that mothers feel that they can provide safe care to the preterm infant after discharge at home. It is encouraged and preferred that all mothers need to receive support after NICU discharge [24], as well as adequate information about BF, support and education, and continuous follow-ups following discharge home [13,18]. It is important that the nurses in the NICU and KMC unit have the requisite skills and understand the significant role they play in the mothers’ confidence in their ability to breastfeed [20].

This study is not without limitations. FGDs were considered an ideal method in the current study to elicit mothers’ description on the BF intentions and practices of mothers of VLBW infants at home following discharge and to further assess the role that long stay in KMC had on their decision to BF beyond discharge. However, the use of FGDs might have allowed the tendency for certain types of socially acceptable opinions to emerge and for certain types of participants to dominate the discussion, among other limitations. The study examined the mothers’ intentions to BF and EBF after discharge, and the fact that we did not follow the mothers up after discharge to check how much the motivation to BF and EBF translated into real BF is a limitation. Whereas this study has been able to attain its objective on describing the BF intentions and practices of mothers of VLBW infants at home following discharge, it is crucial that additional research be conducted to investigate BF after discharge to identify enablers and barriers to sustain BF after discharge. 

## 5. Conclusions

The stay in KMC had a positive influence on the mothers’ intentions not only to breastfeed their preterm infants but also to exclusively breastfeed for a duration of six months. The mothers had positive feelings toward BF their preterm infants, valued the benefits of BF to their infants, and had clear intentions to continue BF at home after discharge. 

Confidence and BF efficacy were influenced to a large extent by training and support that the nurses provided for mothers as they transitioned from tube feeding to direct BF during their stay in the KMC unit. 

It is important that nurses in these settings recognise the individual mother’s needs during discharge planning. Furthermore, they should be cognisant of the role discharge planning plays to increase a mother’s readiness, BF success, and baby’s transition from hospital to home. 

## Data Availability

The data that support the findings of this study are available from the corresponding author, upon reasonable request.
